# Soluble toll-like receptor 4 reversed attenuating effect of Chinese herbal Xiao-Qing-Long-Tang on allergen induced nerve growth factor and thymic stromal lymphopoietin

**DOI:** 10.3892/etm.2013.1294

**Published:** 2013-09-13

**Authors:** REN-SHIU CHANG, YU-CHIN WANG, SHUNG-TE KAO

**Affiliations:** 1 Graduate Institute of Chinese Medicine, China Medical University, Taichung 40402;; 2 Department of Chinese Medicine, Tainan Sin-Lau Hospital, Tainan 70142;; 3 School of Chinese Medicine, College of Chinese Medicine, China Medical University, Taichung 40402;; 4 Department of Chinese Medicine, China Medical University Hospital, Taichung 40447, Taiwan, R.O.C.

**Keywords:** Xiao-Qing-Long-Tang, nerve growth factor, thymic stromal lymphopoietin, soluble Toll-like receptor 4

## Abstract

Xiao-Qing-Long-Tang (XQLT) is known to regulate allergic immune reactions. The aim of this study was to investigate the effects of XQLT on allergen-induced cytokines and associated signaling pathways. An acute allergic mouse model was used to investigate the effects of XQLT on nerve growth factor (NGF) during an allergic reaction, while human pulmonary alveolar epithelial cells (HPAEpiCs) were used to investigate the effects of XQLT on *Dermatophagoides pteronyssinus* group 2 (Der p 2)-induced NGF, p75 neurotrophin receptor (p75NTR) and thymic stromal lymphopoietin (TSLP) expression. XQLT was demonstrated to inhibit NGF- and p75NTR-related allergic reactions in the mouse model. XQLT also reduced the expression of Toll-like receptor 4 (TLR4) in the lungs of the model mice. XQLT inhibited Der p 2-induced NGF, TSLP and p75NTR expression in the HPAEpiC cell line. The use of recombinant soluble TLR4 (sTLR4) in a competitive assay partially attenuated the inhibitory effect of XQLT on NGF, TSLP and p75NTR expression in HPAEpiC cells. The inhibitory effect of XQLT on the Ser536 phosphorylation of p65 (nuclear factor-κB; NF-κB), a TLR4-induced factor, was also attenuated by sTLR4. In conclusion, XQLT inhibited Der p allergen-induced NGF, p75NTR and TSLP expression. This inhibition was attenuated by sTLR4. The mechanism of action of XQLT may be correlated with TLR4, a primary receptor in the innate immune system. The findings of this study may focus the search for pharmacological targets of XQLT onto TLR4, which is important in the allergen presentation pathway.

## Introduction

Traditional Chinese Medicine (TCM), such as Xiao-Qing-Long-Tang (XQLT), is frequently used in Asia for the clinical treatment of bronchial allergic asthma and allergic rhinitis (1,2). It has beneficial effects in relieving allergic inflammation reactions in the airways of allergic animal models (3–6). XQLT is highly popular in TCM and complementary medicine. The identification of potential pharmacological targets of XQLT may provide a basis for evaluating the effectiveness of XQLT in controlling allergic reactions.

Allergen-induced epithelial-origin nerve growth factor (NGF) has been demonstrated to be involved in the early phase of allergic reactions (7–11). NGF, a member of the neurotrophin family, is a product of activated Th2 cells (7,8) and is expressed in multiple cells, including epithelial cells. In allergy, the tissue that is primarily responsible for allergen presentation is the epithelium. Epithelial cells present allergens and induce allergy pathways involving numerous events, including dendritic cell activation and chemokine secretion (12,13). Moreover, NGF has been observed at an elevated concentration in patients with allergic diseases. Toll-like receptor 4 (TLR4), the primary signal that is involved in the early phase of allergic signal transduction (14), is activated by *Dermatophagoides pteronyssinus* (Der p) allergen (15–17), leading to epithelial thymic stromal lymphopoietin (TSLP) expression (18). TSLP has been shown to have a central function in the response to allergen-stimulated TLR4 signals in lung epithelial cells (19–21). The TLRs form a large protein family that are apparent in innate immunity and are responsible for presenting antigens or allergens, such as lipopolysaccharide (LPS) or Der p (22,23). The signaling pathways under TLR4 are relatively complicated. The major factors responsible for TLR4 signal transduction are MyD88 and nuclear factor-κB (NF-κB) (24,25). It has been demonstrated that a TLR4 signal may lead to the Ser536 phosphorylation of the p65/RelA subunit of NF-κB. Following phosphorylation, NF-κB translocates into the nucleus to act as a transcriptional factor.

TLR4 induces NGF and the low-affinity NGF receptor, p75 neurotrophin receptor (p75NTR), in dendritic cells (DCs) (26). *Dermatophagoides pteronyssinus* group 2 (Der p 2) has been demonstrated to have a structural homology with MD-2, the LPS-binding component of the TLR4 complex (27,28). Moreover, a recent study by this team demonstrated that Der p 2 induced NGF production and reactive oxygen species in the airway, as well as allergic inflammation, following direct intratracheal (i.t.) instillation into the lungs of mice (27). The p75NTR is a low-affinity receptor for all factors of the neurotrophin family (29,30). Allergic inflammation and eosinophil infiltration have been eliminated in p75NTR-knockout mice (29,30). p75NTR is known for inducing NF-κB activation, which has been demonstrated to be a major transcriptional factor in the Th2-type immune response (31,32). NGF may also affect DCs through p75NTR (33).

This study showed that the oral administration of XQLT to mice reduced the levels of p75NTR and TLR4 expression in the lungs of allergic mice. In a HPAEpiC cell line, adding exogenous recombinant soluble TLR4 (sTLR4) or a rough strain Salmonella LPS (Re 595) attenuated the XQLT-mediated suppression of Der p 2-stimulated NGF, p75NTR and TSLP expression. Adding sTLR4 also attenuated the inhibitory effect of XQLT on the Ser536 phosphorylation of NF-κB. These results revealed that the inhibitory effect of XQLT may be correlated with TLR4. The study provides evidence useful in further investigations into the pharmacological targets of XQLT in the treatment of allergic reactions.

## Material and methods

### TCM preparation: XQLT

XQLT was obtained and prepared as previously described (34). Briefly, eight crude plant ingredients were mixed: Pinellia tuber (6.0 g, root of *Pinellia ternata* Breitenbach), Ephedrae herba (3.0 g, stem of *Ephedra sinica* Stapf), Schizandrae fructus (3.0 g, a fruit of *Schizandra chinensis* Baill), Cinnamonomi cortex (3.0 g, cortex of *Cinnamomum cassia* Blume), Paeoniae radix (3.0 g, root of *Paeonia lactiflora Pall.*), Asariherba cum radice (3.0 g, whole plant of *Asiasarum heterotropoides* F. Maekawa var. *mandshuricum* F. Maekawa), Glycyrrhizae radix (2.0 g, root of *Glycyrrhiza uralensis* Fisch. Et. DC) and Zingiberis siccatum rhizoma (1.0 g, steamed root of *Zingiber officinale* Roscoe). The mixture was extracted sequentially with 17.5 l and 12.5 l boiling water for 1 h each time, prior to the extracted liquid being mixed and filtered. Following filtration, the dregs of the decoction were removed. The filtered liquid was subsequently lyophilized and crushed into a fine powder. The yield of dried extract from the crude starting material was 661.8 g (26.4%, w/w). The batch number was 98041021.

The XQLT mixture was suspended in distilled water at a fixed concentration for oral administration to the mice, through a feeding needle and swallowing. The feeding volume was adjusted within 0.2–0.3 ml to avoid the mice experiencing any pain. For *in vitro* use, the XQLT mixture was dissolved in distilled water and the solution was subsequently centrifuged at 4,500 × g. Following filtration, the aqueous extract was lyophilized and weighed. This XQLT extract was then re-dissolved in pyrogen-free isotonic saline (YF Chemical Corp., Taipei, Taiwan) and filtered through a 0.2 mm filter (Microgen, Laguna Hills, CA, USA). A sample of the filtered pyrogen-free solution was lyophilized and weighed, prior to the final concentration of the filtered pyrogen-free solution in the sample being estimated. The filtered pyrogen-free solution was stored at −20°C as a stock solution.

### Acute allergic mouse model

Specific pathogen-free, 6–8-week-old BALB/c mice from the Laboratory Animal Center of the National Science Council (Taiwan, R.O.C.) were used in this study. The mice were housed in microisolator cages (Laboratory Products, Maywood, NJ, USA) and provided with sterile food and water. The care and treatment of the experimental animals followed the guidelines of the National Science Council of the Republic of China. The protocol was approved by the Institutional Animal Care and Use Committee (IACUC) of China Medical University (Permit No. 101–159-N; Taichung, Taiwan). All efforts were made to minimize suffering.

**Figure 1.**
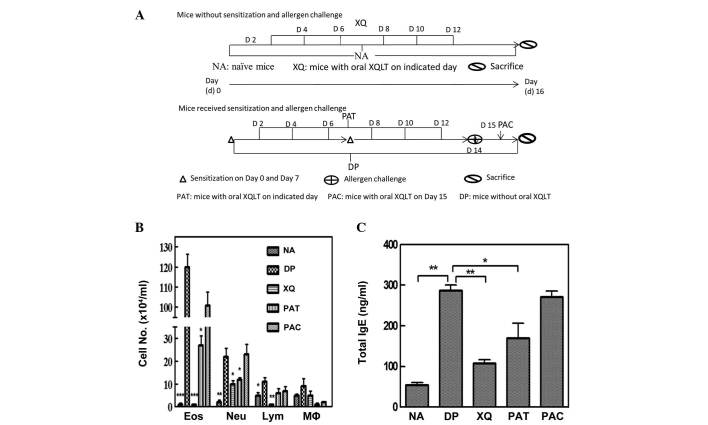
Xiao-Qing-Long-Tang (XQLT) inhibited allergic inflammation in an acute *Dermatophagoides pteronyssinus* (Der p)-challenged mouse model. (A) Schematic of the complete protocol for XQLT treatment. (B) Differential cell counting in broncho-alveolar lavage fluids (BALF) showed that XQLT inhibited inflammation (PAT and PAC). (C) Enzyme-linked immunosorbent assay (ELISA) showed that XQLT decreased the level of total immunoglobulin (Ig) E in the serum (PAT and PAC). Values represent the mean ± standard deviation; n=6 mice for each group. P<0.01, statistics of all groups were versus the mice without oral XQLT (DP group). Eos, eosinophils; Neu, neutrophils; Lym, lymphocytes; Mϕ, macrophages. ^*^P<0.05, ^**^P<0.01, ^***^P<0.005.

The establishment of the acute allergic mouse model was based on a previous study (3) and comprised two stages, the sensitization and the acute allergy induction stages. In the sensitization stage, the mice were administered with a subcutaneous injection at the base of the tail of 50 ml Der p (1 mg/ml) emulsified in incomplete Freund’s adjuvant (IFA; Difco Laboratories, Inc, Detroit, MI, USA); one further boosting injection was administered with the same dose of Der p on day seven. Fourteen days later, the acute allergy induction stage was initiated. The mice were lightly anesthetized with an intraperitoneal (i.p.) injection of 60 mg/kg body weight (BW) sodium pentobarbital (Nembutal; Abbott Laboratories, Chicago, IL, USA). Following this, an i.t. instillation of 50 ml Der p (2 mg/ml) was administered to the mice as an allergen challenge (AC). Acute Der p allergen-challenged mice, which comprised the DP group, were fully established following this protocol. To evaluate the effects of XQLT on this model, mice in the post-allergy curing protocol group (PAC) were treated with 1 g/kg BW XQLT once, 24 h subsequent to the AC. The mice in the pre-allergy treatment protocol group (PAT) were treated with 1 g/kg BW XQLT every two days, from day two until day 12, a total of six times, prior to the AC on day 14. Mice in the control group (XQ) were treated with 1 g/kg BW XQLT every two days, from day two until day 12, a total of six times, without the AC on day 14. The naive mice group (NA) consisted of animals without Der p sensitization, challenge or XQLT treatment. All the mice were sacrificed on day 16. The XQLT dose was based on the results of our previous investigation (3). Each group comprised six mice. The groups are schematically depicted in Fig. 1A.

### Collection of BALF and serum

The mice were sacrificed by administering an overdose of sodium pentobarbital (20 mg/ml) subsequent to the Der p challenge. Following the sacrifice, BALF was collected by flushing the lungs with two separate saline washes, through the trachea, with ~1 ml BALF recovered each time. The BALF samples were centrifuged at 200 × g for 5 min at 4°C to collect the cells. The collected cells were subsequently washed with red blood cell lysis solution and were diluted with RPMI-1640 medium (Gibco-BRL, Invitrogen Life Technologies, Inc., Gaithersburg, MD, USA). The total leukocyte content of the BALF was determined by cytometry to be 1×10^5^ cells/ml. Blood was collected either through the axillary artery or directly from the heart. The collected blood was left to stand for 1 h at room temperature to clot. Centrifugation at 18,000 × g removed the clotted matter to leave the serum.

### Total immunoglobulin (Ig) E concentration in the serum

The IgE concentration in the serum was measured using an enzyme-linked immunosorbent assay (ELISA) kit (Mouse IgE ELISA Quantitation Set, cat. no. E90–115; Bethyl Laboratories, Inc., Montgomery, TX, USA), in accordance with the manufacturer’s instructions. Absorbance was measured at a wavelength of 450 nm on an ELISA plate reader.

### Differential cell counting in the BALF

BALF cells were spun down onto a glass slide at 360 rpm for 8 min by cytospinning. Then, the slides were dried and stained by eosinophil-specific staining methods (Eosinophil-Mast Cell Stain kit, CEM-1-IFU; ScyTek Laboratories, Inc., Logan, UT, USA). A total of >200 cells were counted under a photomicroscope to determine the numbers of lymphocytes, macrophages, neutrophils and eosinophils, as percentages of total BALF cells.

### Immunohistochemistry of p75NTR/TLR4 in lung slices

The paraffin-embedded lung slices were mounted on glass slides, and the paraffin was then depleted away at 60°C. The slices were treated using a number of reagents in the following sequence: xylene, ethanol, 3% H_2_O_2_ (80% methanol) (v/v), and 0.01 M sodium citrate buffer (pH 6.0, 95°C). Following this, 10% non-fat milk was used to block the cooled slices, and rabbit polyclonal anti-p75NTR antibody (Abcam plc, Cambridge, UK) or rabbit anti-TLR4 antibody (Abcam plc) was used for immunostaining (4°C, overnight). Anti-rabbit IgG antibody [fluorescein isothiocyanate (FITC) or phycoerythrin-conjugated; Abcam plc] was used as a secondary antibody to develop fluorescence. The developed slice was observed under a light microscope and the area density of fluorescence was analyzed using an inverted fluorescence microscope (Olympus IX71^®^; Olympus, Center Valley, PA, USA) and VisionWorks®LS Analysis Software (UVP, Upland, CA, USA). The results concerning fluorescence density are presented as a bar graph.

### NGF and TSLP production in human pulmonary alveolar epithelial cells following Der p 2 stimulation

The human lung epithelial cell line, HPAEpiC, comprising alveolar type I and type II epithelial cells, was purchased from Sciencell Research Laboratories (Carlsbad, CA, USA). HPAEpiC cells were cultured in Dulbecco’s modified Eagle medium (DMEM) F12 medium (Gibco-BRL), containing 10% fetal bovine serum (FBS) and 1% penicillin/streptomycin, in a lysine-coated flask in an incubator at 37°C with 5% CO_2_.

Table I (XQLT dose-response) and Table II (TLR4 competitive assay) show the experimental groups that were established. The term ‘XQLT pulse 1 h’ referred to the cells being treated with XQLT for 1 h, prior to the XQLT being washed out. Der p 2 was then used to stimulate the cells pretreated with XQLT for the following 24 h. Supernatants was collected for NGF and TSLP ELISA analysis, respectively. Total protein was extracted from the treated cells. The LPS source was *Salmonella minnesota* Re 595 (cat. no. L-9764; Sigma-Aldrich, St. Louis, MO, USA), a known TLR4 ligand (35). The commercially available sTLR4 was purchased from USCN^®^ Life Science Inc. (Houston, TX, USA). Prior to treatment with XQLT, all cells underwent starvation (serum-free status) for ≥8 h. Whenever the cells were treated with XQLT or Der p 2, 1% FBS was added to the cell culture to maintain cell survival.

ELISA was used to analyze the concentrations of NGF and TSLP. The concentration of NGF in the BALF or cell culture supernatant was measured using an ELISA kit (NGF Emax^®^ ImmunoAssay Systems; Promega Corporation, Madison, WI, USA), in accordance with the manufacturer’s instructions. The concentration of TSLP in the cell culture supernatant was also measured using an ELISA kit (TSLP: Human TSLP ELISA Ready-SET-Go^®^; eBioscience, Inc., San Diego, CA, USA), in accordance with the manufacturer’s instructions.

### Western blot analysis of the levels of p75NTR and Ser536 phosphorylated p65 (NF-κB)

Cells (1–2 × 10^6^ cells/ml) were lysed using a Triton X-100-based lysis buffer that contained 1% Triton X-100, 150 mM NaCl, 10 mM Tris (pH 7.5), 5 mM EDTA, 5 mM NaN_3_, 10 mM NaF and 10 mM sodium pyrophosphate. Cell extracts were separated using sodium dodecyl sulfate-polyacrylamide gel electrophoresis (SDS-PAGE), prior to being transferred to a polyvinylidene difluoride (PVDF) membrane (Millipore, Billerica, MA, USA). Subsequent to blocking, the blots were developed using a rabbit polyclonal anti-p75NTR antibody (cat. no. ab8874, Abcam plc) or rabbit polyclonal anti-phospho-NF-κB p65 (Ser536) antibody (cat. no. 3031; Cell Signaling Technology, Inc., Danvers, MA, USA). The blots were subsequently hybridized using horseradish peroxidase (HRP)-conjugated goat anti-rabbit IgG (Calbiochem, San Diego, CA, USA) and developed with a chemiluminescence kit (Western Lightning™ Chemiluminescence Reagent Plus; PerkinElmer Life Sciences Inc., Boston, MA, USA). The optical density that corresponded with the p75NTR or Ser536 phosphorylated p65 to total protein ratio was determined using an image analysis system.

### Statistical analysis

The data are expressed as the mean ± standard deviation. Statistical comparisons were performed using the Student’s t-test, with P<0.05 or P<0.01 considered to indicate a statistically significant difference. All statistics are indicated in the figure legends.

## Results

### Differential cell counting in BALF and total IgE production demonstrate that XQLT inhibits allergic inflammation

The effect of XQLT on allergic inflammation was studied in an established acute Der p-stimulated allergic mouse model (3). Fig. 1A shows the experimental animal groups. Der p-stimulated mice that had been orally treated with XQLT (PAT and PAC; Fig. 1B) had fewer infiltrating cells in the BALF compared with the Der p-challenged mice without oral XQLT administration (DP; Fig. 1B). Further analysis showed that the oral administration of XQLT to mice, particularly as a pre-allergy treatment, reduced total IgE production (PAT; Fig. 1C). In addition, XQLT was demonstrated to reduce allergic inflammation, particularly when administered orally as a pre-allergy treatment (PAT; Fig. 1B and C). The mice with XQLT treatment alone (XQ) did not exhibit higher cell infiltration or total IgE production than the NA group. Therefore, XQLT was shown to suppress the allergic reaction in the acute Der p-stimulated mouse model.

### XQLT reduces NGF, p75NTR and TLR4 expression in the lungs

**Figure 2.**
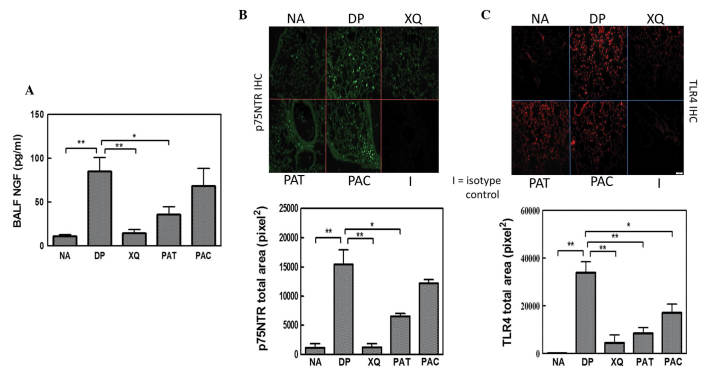
Enzyme-linked immunosorbent assay (ELISA) and immunohistochemistry showing the inhibitory effects of Xiao-Qing-Long-Tang (XQLT) on *Dermatophagoides pteronyssinus* (Der p)-induced nerve growth factor (NGF)/p75 neurotrophin receptor (p75NTR)/Toll receptor-like 4 (TLR4) expression in the lungs. Der p allergen significantly induced NGF/p75NTR/TLR4 expression in the lungs in the the allergen-challenged mice without oral XQLT (DP group). XQLT oral administration, particularly as a preventative strategy (PAT group), decreased (A) NGF, (B) p75NTR and (C) TLR4 expression. The average area of fluorescence density in immunohistochemistry was calculated from the fluorescence of 36–50 vision fields in each group. Values represent the mean ± standard deviation; n=6 mice for each group. P<0.01, statistics of all groups were versus group DP. NA, naive mice; XQ, mice with XQLT treatment, but without allergen challenge; PAC, post-allergy curing protocol group; BALF, broncho-alveolar lavage fluids. ^*^P<0.05, ^**^P<0.01.

In the preceding section, XQLT was demonstrated to inhibit allergic reaction in a mouse model. NGF promotes the TLR4 signaling-induced maturation of DCs through inducible p75NTR, an important event in allergy initiation (33). ELISA analysis showed that the oral administration of XQLT to mice, particularly pre-allergically (PAT; Fig. 2A) reduced NGF expression in the BALF. Immunohistochemistry revealed that Der p stimulated strong p75NTR expression in the lungs of mice (DP; Fig. 2B). The majority of the Der p-induced NGF receptors were observed around the epithelia. XQLT treatment reduced the Der p-induced p75NTR expression in the lungs, particularly in the mice that had received a pre-allergy oral administration of XQLT (PAT; Fig. 2B). The XQ mice (oral XQLT alone) did not exhibit a greater expression of p75NTR than the NA mice. XQLT appeared to downregulate the Der p-induced NGF and p75NTR expression simultaneously. It is speculated that XQLT downregulated NGF and p75NTR expression through an upstream pathway and that TLR4 may be one of the targets of XQLT.

Immunohistochemistry showed that Der p stimulated marked TLR4 expression in the lungs of mice (DP; Fig. 2C). The majority of the expressed TLR4 was focused around the epithelium. The pre- and post-allergy oral administration of XQLT to mice reduced the expression of TLR4 in the Der p-stimulated lung. The XQLT-inhibited TLR4 expression was not fully consistent with the XQLT-inhibited NGF receptor expression, particularly in the PAC mice. The relationship between the inhibitory effects of XQLT on TLR4 and NGF receptor expression remains unclear.

XQLT appeared to simultaneously inhibit the expression of NGF and p75NTR in the lung. To study the effects of XQLT on NGF and its receptors, Der p 2, a known inducer of NGF and the TLR4 signal, was used to stimulate an XQLT-treated cell line that originally morphologically and physiologically resembled lung cells.

### XQLT pretreatment reduced the Der p 2-induced expression of NGF and TSLP in the cell line model

**Figure 3.**
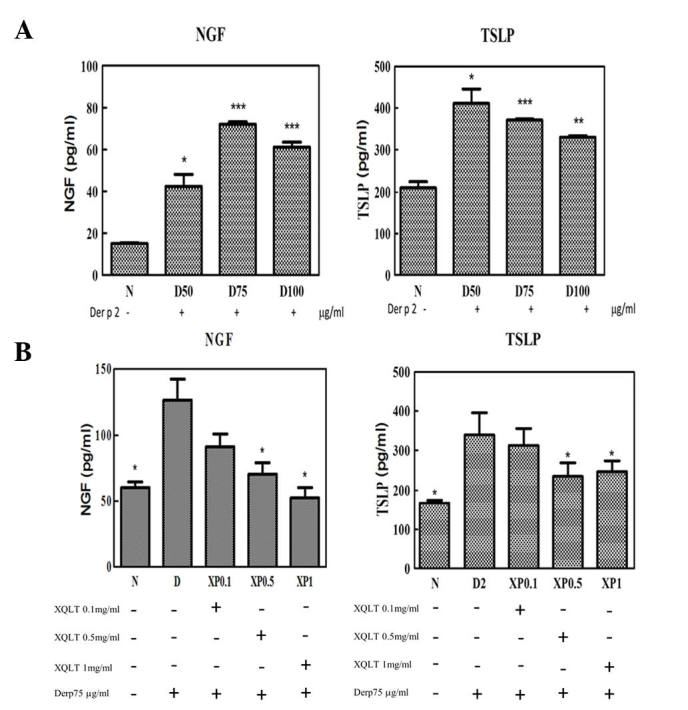
Effects of Xiao-Qing-Long-Tang (XQLT) on a *Dermatophagoides pteronyssinus* group 2 (Der p 2)-stimulated cell line model. (A) Der p 2 induced nerve growth factor (NGF) and thymic stromal lymphopoietin (TSLP) expression in the HPAEpiC cell line model with a concentration-dependent response. (B) XQLT decreased the Der p 2-induced NGF and TSLP expression in the HPAEpiC cell line model with a concentration-dependent response (XP0.1~XP1). Values represent the mean ± standard deviation. P<0.01, statistics of all groups were versus the cells stimulated with Der p 2 alone (D). Each test is triplicate and was repeated at least three times. ^*^P<0.05, ^**^P<0.01, ^***^P<0.005.

To evaluate the effects of XQLT on the Der p 2 signaling pathway in a cell line model, the expression of NGF, p75NTR and TSLP was tested. HPAEpiC, a human cell line comprising type I and type II alveolar cells, was used to evaluate the ability of Der p 2 to induce the expression of NGF, p75NTR and TSLP *in vitro*. Der p 2 exhibited a concentration-dependent ability to induce the expression of NGF and TSLP in the HPAEpiC cell line (Fig. 3A). Table I shows the experimental design used to study the effect of varying XQLT concentrations on the Der p 2-induced expression of NGF, TSLP and p75NTR in the cell line model. Briefly, XQLT was used to pretreat the cells for 1 h, prior to the XQLT being washed out. Cells were subsequently stimulated with Der p 2 for 24 h. Pretreatment with XQLT reduced the expression of NGF and TSLP in the Der p 2-stimulated cell line, with a concentration-dependent response (Fig. 3B). Furthermore, western blot analysis showed that pretreatment with XQLT inhibited the expression of p75NTR in Der p 2-stimulated cell lines (data not shown). All the results demonstrated that XQLT inhibited Der p 2 signaling, indicating the inhibition of TLR4 and its downstream signaling.

### Adding sTLR4 and rough strain Salmonella LPS into cell lines attenuates the reduction in NGF and TSLP expression by XQLT

To study the correlation between XQLT and the TLR4 signaling pathway, a competitive assay was performed to evaluate whether it was possible to attenuate the inhibitory effect of XQLT on the expression of NGF and TSLP. Table II shows in detail all the relevant protocols (TLR4 competitive assay). A TLR4 signaling rough strain of *Salmonella* LPS (Re 595) and exogenous recombinant sTLR4 were used in the competitive assay.

**Figure 4.**
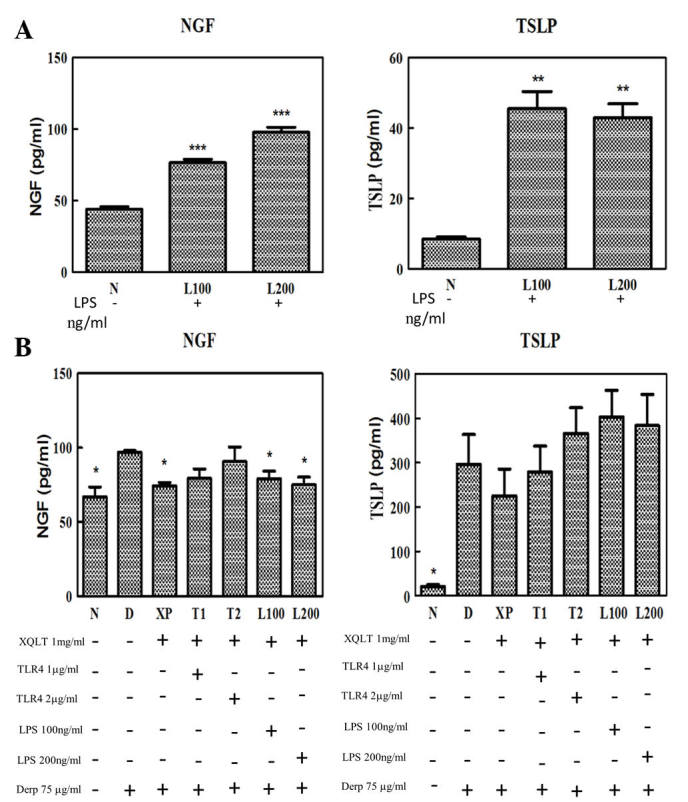
Enzyme-linked immunosorbent assay (ELISA) analysis showed that: (A) lipopolysaccharide (LPS) induced nerve growth factor (NGF) and thymic stromal lymphopoietin (TSLP) expression in HPAEpiC cells; (B) adding recombinant exogenous soluble Toll receptor-like 4 (sTLR4; T1 and T2) attenuated the Xiao-Qing-Long-Tang (XQLT)-inhibited NGF and TSLP expression in *Dermatophagoides pteronyssinus* group 2 (Der p 2)-stimulated HPAEpiC cells; however, adding LPS (L100 and L200) attenuated only the inhibitory effect of XQLT on TSLP expression. Values represent the mean ± standard deviation. P<0.05, statistics of all groups were versus the cells stimulated with Der p 2 alone (D). Each test is triplicate and was repeated at least three times. ^*^P<0.05, ^**^P<0.01, ^***^P<0.005.

XQLT inhibited the expression of NGF and TSLP in the Der p 2-stimulated HPAEpiC cells (XP; Figs. 3B and 4B). Although the *Salmonella* LPS induced the production of NGF in the 24 h HPAEpiC cell culture (Fig. 4A), LPS (L100 and L200; Fig. 4B) did not attenuate the inhibitory effect of XQLT on NGF expression (XP; Fig. 4B) in the Der p 2-stimulated HPAEpiC cells. All statistical calculations were performed with reference to the data from the cells stimulated with Der p 2 alone (D; Fig. 4). By contrast, LPS (L100 and L200) was able, to a certain degree, to attenuate the inhibitory effect of XQLT on TSLP expression (XP). LPS was therefore demonstrated to exhibit a differential effect on the XQLT-mediated inhibition of NGF and TSLP expression.

Recombinant sTLR4 (T1 and T2; Fig. 4B) attenuated the inhibitory effect of XQLT on Der p 2-stimulated NGF and TSLP expression. Although the reversal effects of the sTLR4 require further evaluation, it appeared that the mechanism by which sTLR4 attenuated the inhibitory effects of XQLT on Der p 2-stimulated HPAEpiC cells involved a specific ligand competing effect, although not a nonspecific massive exogenous protein competing effect. To further analyze the correlation between XQLT and the TLR4 pathway, western blot analysis was used to study the p75NTR and Ser536 phosphorylated p65 (NF-κB) in a competing assay.

### XQLT inhibits p75NTR and Ser536 phosphorylation of p65 (NF-κB) and the inhibition is attenuated by sTLR4

**Figure 5.**
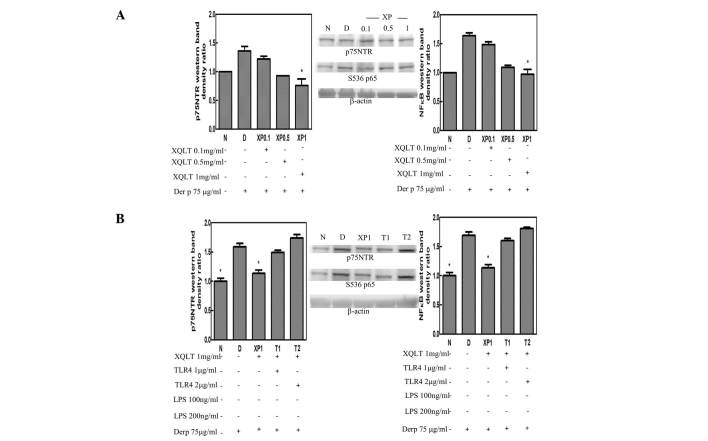
Western blot analysis showed that: (A) Xiao-Qing-Long-Tang (XQLT; XP0.1~XP1) decreased the *Dermatophagoides pteronyssinus* group 2 (Der p 2)-induced p75NTR expression and Ser536 phosphorylation of p65 (nuclear factor-κB; NF-κB) with a concentration-dependent response; (B) adding recombinant exogenous soluble Toll receptor-like 4 (sTLR4; T1 and T2) attenuated the inhibitory effect of XQLT (XP1) on p75NTR expression and Ser536 phosphorylation of p65 (NF-κB). Values represent the mean ± standard deviation. P<0.05, statistics of all groups were versus the cells stimulated with Der p 2 alone (D). Each test is triplicate and was repeated at least three times. ^*^P<0.05.

p75NTR was observed to be induced by a Der p 2 signal, while Ser536 phosphorylation of p65 (NF-κB) is known to be induced specifically by a TLR4 signal. Using steps described in Table I, the pretreatment of HPAEpiC cells with XQLT was observed to inhibit p75NTR and Ser536 phosphorylation of p65 (NF-κB) in concentration-dependent manner (XP0.1~XP1; Fig. 5A). Results of the western blotting were analyzed by a densitometer and are presented as a bar graph with the density value of the negative group (N in Fig. 5) set as 1. A concentration of 1 mg/ml XQLT demonstrated the most marked inhibitory effect on p75NTR and Ser536 phosphorylation of p65 (NF-κB). Furthermore, using the steps described in Table II, adding sTLR4 (T1 and T2; Fig. 5B) was shown to attenuate the inhibitory effects of 1 mg/ml XQLT on p75NTR and Ser536 phosphorylation of p65 (NF-κB). The reversal effect of sTLR4 also demonstrated a concentration-dependent response.

## Discussion

In this study, the known ability of XQLT to suppress allergic reactions in allergen-stimulated mice (2–4,34) was further demonstrated (Fig. 1B and C). However, XQLT is a herbal product that comprises a complex mixture of compounds, which presents challenges for studies investigating its pharmacological action. This study further identified the potential TLR4-related pharmacological targets of XQLT action.

TLR4 is a member of the TLR family and is distributed in the lung and intestinal epithelia (36). It is responsible for detecting pathogens and allergens. Der p 2 is one of the allergens with a structure resembling that of the MD-2 in the TLR4 complex. This study showed that the Der p allergen induced NGF and the low-affinity receptor, p75NTR, in the allergic mouse model (DP; Fig. 2A and B), in addition to inducing the expression of TLR4 (DP; Fig. 2C). XQLT was shown to inhibit the Der p-induced expression of NGF, p75NTR and TLR4 in the acute allergic mice model (PAT and PAC; Fig. 2). Der p 2 induced the expression of NGF and TSLP in the HPAEpiC cell line (Fig. 3A) and XQLT was demonstrated to suppress the Der p 2-induced expression of NGF and TSLP in the HPAEpiC cells (XP; Fig. 3B). *Salmonella* LPS (Re 595) (L100 and L200; Fig. 4B), a TLR4 agonist, and sTLR4 (T1 and T2; Fig. 4B) were used in a competitive assay to attenuate the inhibitory effect of XQLT on TSLP expression in the HPAEpiC cells. sTLR4, but not LPS, attenuated the inhibitory effect of XQLT on NGF and TSLP expression in HPAEpiC cells (T1 and T2; Fig. 4B). These results suggest that the active ligand component of XQLT may affect the TLR4 signaling pathway by binding directly to TLR4, but not to CD14 (the LPS-binding moiety in the TLR4 receptor complex) (37). We hypothesize that the effects of XQLT were mediated through TLR4 by different downstream pathways, leading to the inhibition of TSLP and NGF expression. LPS attenuated the effects of XQLT on TSLP more markedly than it attenuated the effects of XQLT on NGF. This may have been due to different time requirements for LPS to attenuate the effects of XQLT on NGF and TSLP, respectively. Alternatively, the pathway used by LPS to attenuate the effects of XQLT on TSLP may have been different to that used to attenuate the XQLT-mediated inhibition of NGF. Taking into consideration that LPS reversed the XQLT-mediated inhibition of TSLP expression, it may be that the signals associated with CD14 were affected by XQLT, thus leading to reduced TSLP expression in HPAEpiC cells.

A previous study demonstrated that Der p 2 induced p75NTR expression through TLR4 (26). The results of the present study, shown in Fig. 5A, further indicated that Der p 2 induced p75NTR expression (D). XQLT was demonstrated to inhibit p75NTR expression in a concentration-dependent manner. To study whether the TLR4 pathway was affected, a western blot analysis of Ser536 phosphorylation of p65 (NF-κB) was performed. The Der p 2-induced Ser536 phosphorylation of p65 (Fig. 5A) was reduced by XQLT, with a concentration-dependent response. TLR4 has been demonstrated to transduce signals through MyD88 and NF-κB (24,25). Furthermore, p75NTR signals have been shown to induce NF-κB (31,32) and XQLT has been demonstrated to reduce NF-κB translocation into the nucleus (34). In consideration of these results, it was suggested that XQLT may inhibit NGF, p75NTR and TSLP through a pathway involving TLR4. Using sTLR4 for a competing assay (protocol in Table II), the inhibitory effects of XLQT on p75NTR and Ser536 phosphorylation of p65 were attenuated (Fig. 5B). This is the first time, to the best of our knowledge, that XQLT has been observed to potentially regulate the early phase of an allergic response.

The inhibitory effects of XQLT on NGF, p75NTR and TSLP may be associated with the TLR4 pathway, thus providing pharmacological targets for investigations into how XQLT affects the early phase of allergic reactions. The present study indicated that XQLT may have the ability to interfere with the allergen-presenting process occurring in the epithelium. Further studies are required to identify how XQLT acts to prevent allergic reactions and to investigate the effects of XQLT on TLR4. This may be beneficial in the extension of XQLT usage into other diseases involving the TLR4 pathway.

In conclusion, XQLT may regulate NGF, p75NTR and TSLP via the TLR4 pathway in the early phase of an allergic reaction. Therefore XQLT may provide a preventive benefit in the control of allergic reaction.

## Figures and Tables

**Table I.** Xiao-Qing-Long-Tang (XQLT) dose-response.

Treatment	Group				
	D	XP0.1	XP0.5	XP1	N
Der p 2 (75 *μ*g/ml^a^) 24 h	+	+	+	+	-
XQLT pulse 1 h	-	0.1 mg/ml	0.5 mg/ml	1 mg/ml	-

a Dose that maximizes nerve growth factor (NGF) production, determined in pre-titration trial. D, positive control; N, negative control; XQLT pulse 1 h, cells were treated with XQLT for 1 h, prior to the XQLT being washed out.

**Table II.** Toll-like-receptor-4 (TLR4) competing assay.

Treatment	Group						
	N	D	XP	L100	L200	T1	T2
Der p 2^a^	-	+	+	+	+	+	+
XQLT^b^	-	-	+	+	+	+	+
Treatment combined with XQLT	-	-	-	LPS 100 ng/ml	LPS 200 ng/ml	+sTLR4 1 *μ*g/ml	+sTLR4 2 *μ*g/ml

a Der p 2 (75 *μ*g/ml) for 24 h;

b Xiao-Qing-Long-Tang (XQLT; 1 mg/ml) pulse 1 h. Briefly, lipopolysaccharide (LPS) or exogenous recombinant soluble TLR4 (sTLR4) was used to pretreat XQLT at room temperature. The mixture of XQLT and sTLR4 or LPS was then added to the cell culture. One hour subsequently, the previously mentioned XQLT mixture and medium were washed-out. Cells were then stimulated with Der p 2 for the next 24 h. The supernatants and total cell protein were collected 24 h later. N, negative control; D, positive control; XP, XQLT treatment.

## Abbreviations:

XQLT: Xiao-Qing-Long-Tang;
sTLR4: soluble Toll-like receptor 4

## References

[1] Kung YY, Chen YC, Hwang SJ (2006). The prescriptions frequencies and patterns of Chinese herbal medicine for allergic rhinitis in Taiwan. Allergy.

[2] Nagai T, Nakao M, Shimizu Y (2011). Proteomic analysis of anti-inflammatory effects of a Kampo (Japanese herbal) medicine ‘Shoseiryuto (Xiao-Qing-Long-Tang)’ on airway inflammation in a mouse model. Evid Based Complement Alternat Med.

[3] Kao ST, Wang SD, Wang JY (2000). The effect of Chinese herbal medicine, xiao-qing-long tang (XQLT), on allergen-induced bronchial inflammation in mite-sensitized mice. Allergy.

[4] Nagai T, Arai Y, Emori M (2004). Anti-allergic activity of a Kampo (Japanese herbal) medicine ‘Sho-seiryu-to (Xiao-Qing-Long-Tang)’ on airway inflammation in a mouse model. Int Immunopharmacol.

[5] Ko E, Rho S, Cho C (2004). So-Cheong-Ryong-Tang, tradititional Korean medicine, suppresses Th2 lineage development. Biol Pharm Bull.

[6] Ko E, Rho S, Lee EJ (2004). Traditional Korean medicine (SCRT) modulate Th1/Th2 specific cytokine production in mice CD4^+^ T cell. J Ethnopharmacol.

[7] Ehrhard PB, Erb P, Graumann U, Otten U (1993). Expression of nerve growth factor and nerve growth factor receptor tyrosine kinase Trk in activated CD4-positive T-cell clones. Proc Natl Acad Sci USA.

[8] Ehrhard PB, Erb P, Graumann U, Schmutz B, Otten U (1994). Expression of functional trk tyrosine kinase receptors after T cell activation. J Immunol.

[9] Glaab T, Hoymann HG, Hecht M (2003). Effect of anti-nerve growth factor on early and late airway responses in allergic rats. Allergy.

[10] Abram M, Wegmann M, Fokuhl V (2009). Nerve growth factor and neurotrophin-3 mediate survival of pulmonary plasma cells during the allergic airway inflammation. J Immunol.

[11] Shi Y, Jin Y, Guo W (2012). Blockage of nerve growth factor modulates T cell responses and inhibits allergic inflammation in a mouse model of asthma. Inflamm Res.

[12] Hahn C, Islamian AP, Renz H, Nockher WA (2006). Airway epithelial cells produce neurotrophins and promote the survival of eosinophils during allergic airway inflammation. J Allergy Clin Immunol.

[13] Noga O, Peiser M, Altenähr M (2007). Differential activation of dendritic cells by nerve growth factor and brain-derived neurotrophic factor. Clin Exp Allergy.

[14] Phipps S, Lam CE, G, Kaiko GE (2009). Toll/IL-1 signaling is critical for house dust mite-specific helper T cell type 2 and type 17 responses. Am J Respir Crit Care Med.

[15] Hammad H, Chieppa M, Perros F (2009). House dust mite allergen induces asthma via Toll-like receptor 4 triggering of airway structural cells. Nat Med.

[16] Ricci A, Greco S, Mariotta S (2001). Neurotrophins and neurotrophin receptors in human lung cancer. Am J Respir Cell Mol Biol.

[17] Molloy NH, Read DE, Gorman AM (2011). Nerve growth factor in cancer cell death and survival. Cancers.

[18] Liu YJ, Soumelis V, Watanabe N (2007). TSLP: an epithelial cell cytokine that regulates T cell differentiation by conditioning dendritic cell maturation. Annu Rev Immunol.

[19] Al-Shami A, Spolski R, Kelly J (2005). A role for TSLP in the development of inflammation in an asthma model. J Exp Med.

[20] Liu YJ (2006). Thymic stromal lymphopoietin: master switch for allergic inflammation. J Exp Med.

[21] Roan F, Bell BD, Stoklasek TA (2012). The multiple facets of thymic stromal lymphopoietin (TSLP) during allergic inflammation and beyond. J Leukoc Biol.

[22] Lu YC, Yeh WC, Ohashi PS (2008). LPS/TLR4 signal transduction pathway. Cytokine.

[23] Kenny EF, O’Neill LA (2008). Signalling adaptors used by Toll-like receptors: an update. Cytokine.

[24] Kobayashi T, Takaesu G, Yoshimura A (2006). Mal-function of TLRs by SOCS. Nat Immunol.

[25] Wang J, Cai Y, Shao LJ (2011). Activation of NF-κB by TMPRSS2/ERG fusion isoforms through Toll-like receptor-4. Cancer Res.

[26] Jiang Y, Chen G, Zheng Y (2008). TLR4 signaling induces functional nerve growth factor receptor p75NTR on mouse dendritic cells via p38MAPK and NF-κB pathways. Mol Immunol.

[27] Ye YL, Wu HT, Lin CF (2011). *Dermatophagoides pteronyssinus* 2 regulates nerve growth factor release to induce airway inflammation via a reactive oxygen species-dependent pathway. Am J Physiol Lung Cell Mol Physiol.

[28] Trompette A, Divanovic S, Visintin A (2009). Allergenicity resulting from functional mimicry of a Toll-like receptor complex protein. Nature.

[29] Tokuoka S, Takahashi Y, Masuda T, Tanaka H, Furukawa S, Nagai H (2001). Disruption of antigen-induced airway inflammation and airway hyper-responsiveness in low affinity neurotrophin receptor p75 gene deficient mice. Br J Pharmacol.

[30] Kerzel S, Päth G, Nockher WA, Quarcoo D, Raap U, Groneberg DA, Dinh QT, Fischer A, Braun A, Renz H (2003). Pan-neurotrophin receptor p75 contributes to neuronal hyper-reactivity and airway inflammation in a murine model of experimental asthma. Am J Respir Cell Mol Biol.

[31] Bothwell M (1996). p75NTR: a receptor after all. Science.

[32] Carter BD, Kaltschmidt C, Kaltschmidt B (1996). Selective activation of NF-κB by nerve growth factor through the neurotrophin receptor p75. Science.

[33] Jiang Y, Chen G, Zhang Y (2007). Nerve growth factor promotes TLR4 signaling-induced maturation of human dendritic cells in vitro through inducible p75NTR. J Immunol.

[34] Wang SD, Lin LJ, Chen CL, Lee SC, Lin CC, Wang JJ, Kao ST (2012). Xiao-Qing-Long-Tang attenuates allergic airway inflammation and remodeling in repetitive *Dermatogoides pteronyssinus* challenged chronic asthmatic mice model. J Ethnopharmacol.

[35] Muroi M, Tanamoto KI (2002). The polysaccharide portion plays an indispensable role in *Salmonella* lipopolysaccharide-induced activation of NF-κB through human toll-like receptor 4. Infect Immun.

[36] Nishimura M, Naito S (2005). Tissue-specific mRNA expression profiles of human toll-like receptors and related genes. Biol Pharm Bull.

[37] Zanoni I, Ostuni R, Marek LR (2011). CD14 controls the LPS-induced endocytosis of Toll-like receptor 4. Cell.

